# Salivary gland carcinoma: Prediction of cancer death risk based on apparent diffusion coefficient histogram profiles

**DOI:** 10.1371/journal.pone.0200291

**Published:** 2018-07-05

**Authors:** Misa Sumi, Takashi Nakamura

**Affiliations:** Department of Radiology and Cancer Biology, Nagasaki University Graduate School of Biomedical Sciences, Nagasaki, Japan; University of Colorado Denver, UNITED STATES

## Abstract

We evaluated apparent diffusion coefficient (ADC) histogram parameters for predicting the outcomes of patients with salivary gland carcinoma. Diffusion-weighted MR imaging was performed in 20 patients with salivary gland carcinoma, and ADCs were determined using b-values of 500 and 1000 s/mm^2^. ADC histogram parameters (mean, median, percentage tumor area with distinctive ADC values [pADC], skewness, and kurtosis) were analyzed. The patients were followed for 5–136 months after primary surgery. The ADC histogram parameters and T (pT), N(pN), and M categories of the primary tumors were assessed for the prognostic importance using Cox proportional hazards models, logistic regression analysis, and receiver operating characteristic (ROC) analysis. Cohen’s d was determined for evaluating the importance of differences in the parameters between two patient groups with different outcomes. Six patients died of cancer (DOC) within 3 years after the primary surgery. Cox proportional hazards models indicated that ADC mean (95% CI = 0.494–0.977, p = 0.034), ADC median (95% CI = 0.511–0.997, p = 0.048), pADC with extremely low (<0.6 mm^2^/s) ADC (95% CI = 1.013–1.082, p = 0.007), kurtosis (95% CI = 1.166–7.420, p = 0.023), and pN classification (95% CI = 1.196–4.836, p = 0.012) were important factors of cancer death risk. ROC analyses indicated that the pADC <0.6 ×10^−3^ mm^2^/s was the best prognostic predictor (p <0.001; AUC = 0.929) among the ADC and TNM classification parameters that were significant in a univariate logistic regression analysis. Cohen’s d values between the DOC and survived patients for the ADC mean, ADC median, pADC with extremely low ADC, and kurtosis were 1.06, 1.04, 2.12, and 1.13, respectively. These results suggest that ADC histogram analysis may be helpful for predicting the outcomes of patients with salivary gland carcinoma.

## Introduction

Salivary gland carcinomas show a striking range of histological diversity between different carcinoma types and within individual tumors [1]. The morphological variability of salivary gland carcinomas makes the diagnosis difficult. Moreover, persistent genomic instability in some types of benign salivary gland tumors may progress to malignancy, further complicating the diagnosis [2]. Histological grades and carcinoma types have been shown to be independent predictors of patient prognosis [3, 4]. However, the preoperative imaging diagnosis of histological types and grades is daunting. Accordingly, increasing demands have been arisen for pre-operative fine-needle aspiration cytology (FNAC). However, in addition to non-diagnostic results of FNAC, variability of immunohistological features of salivary gland carcinomas also makes the classification of histological types and grades difficult [5, 6]. Metastasis to the regional lymph nodes are often associated with salivary gland carcinomas [1], and locoregional neck failure may lead to distant metastasis, further worsening the patient prognosis [7]. Therefore, preoperative prediction of the outcomes of patients with salivary gland carcinoma is daunting.

Recently, the combined use of diffusion-weighted and contrast-enhanced MR imaging techniques has been shown to effectively discriminate malignant from benign salivary gland tumors [8]. To assess the diffusion property of biological tissues, we can use a diffusion parameter, apparent diffusion coefficient (ADC). The parameter is introduced for describing the diffusion process in a human body, where the completely free diffusion process is hindered by the presence of membranes, macromolecules, fibers, and other intracellular and extracellular structures. Sumi et al. showed that intravoxel incoherent motion (IVIM) imaging, which enables a separate assessment of diffusion from perfusion properties of biological tissues, can differentiate between pleomorphic adenomas, Warthin tumors and malignant salivary gland tumors with 100% accuracy without contrast medium [9, 10]. In these studies, percentage tumor area with distinctive ADC values (pADC) was found to be an important indicator of some types of salivary gland tumors. Furthermore, ADC histogram analysis using multiple ADC parameters, including skewness, kurtosis and ADC percentiles, was found to effectively predict the treatment outcomes of patients with head and neck squamous cell carcinomas [11, 12], and the analysis accurately discriminated aggressive prostate cancers [13].

These results prompted us to investigate the usefulness of ADC histogram analysis when preoperatively discriminating salivary gland carcinomas that can lead to poor prognostic outcomes of the patients. Here, we retrospectively analyzed ADC histograms from patients with salivary gland carcinomas and tested the possibility that ADC histogram parameters could predict the risk of cancer death.

## Materials and methods

### Patients

We searched the clinical and imaging databases of the Nagasaki University Hospital and identified 20 patients with histologically proven salivary gland carcinoma who received preoperative conventional and diffusion-weighted magnetic resonance (MR) imaging between May 2003 and December 2011 (13 men and 7 women; mean age, 60 ± 13 years; age range, 33–82 years). Subsequently, all the patients underwent surgical excision of the primary tumors with or without neck dissections for the metastatic node(s). The exclusion criteria were (1) preceding or overlapping malignancies and (2) primary recurrence. All the patients were followed for 5–136 months after surgical excision of the primary lesion. During these periods, 6 out of the 20 patients died of cancer resulting from distant metastasis and/or local failure within the first 3 years (5–18 months) after the primary surgery (**Table 1**); the other 14 patients with or without local failure and/or distant metastasis had survived beyond the first 3 years (42–136 months) from the primary surgery.

**Table 1:** Histological subtypes and ADC parameters of 20 salivary gland carcinomas.

subtype (n)	mean	median	skewness	kurtosis	pADC (percentage tumor area with)				DOC¶
	x10^-3^mm^2^/s	x10^-3^mm^2^/s			<0.6 ADC	0.6≤ADC<1.2	1.2≤ADC<1.8	≥1.8 ADC	
CxPA (4)	1.23 ± 0.19	1.18 ± 0.20	0.22 ± 0.70	0.58 ± 0.46	4.9 ± 5.8	46.1 ± 24.0	39.4 ± 16.4	9.7 ± 8.7	0
ACCa (4)	1.03 ± 0.22	1.02 ± 0.22	0.06 ± 0.30	0.24 ± 0.63	6.8 ± 7.0	66.0 ± 29.5	23.9 ± 26.0	3.3 ± 4.7	1
EMC (2)	1.56 ± 0.13	1.40 ± 0.04	0.39 ± 0.05	-0.69 ± 0.99	0.5 ± 0.4	29.5 ± 9.7	42.7 ± 25.8	27.3 ± 15.7	0
SDC (2)	0.78 ± 0.23	0.77 ± 0.27	0.44 ± 0.27	0.44 ± 0.24	30.5 ± 31.4	61.4 ± 25.3	8.1 ± 6.2	0 ± 0	2
ANOS (3)	0.90 ± 0.11	0.82 ± 0.04	0.82 ± 0.76	1.32 ± 1.66	14.5 ± 10.1	70.8 ± 20.7	9.5 ± 9.9	5.3 ± 6.8	1
MEC (3)	0.90 ± 0.12	0.88 ± 0.13	0.13 ± 0.64	0.88 ± 0.68	14.4 ± 18.8	76.5 ± 25.1	7.9 ± 5.0	1.2 ± 2.0	1
LEC (1)	0.77	0.74	0.49	-0.59	10.4	89.6	0	0	0
SCCa (1)	0.44	0.38	0.83	0.81	72.3	25.5	2.2	0	1

CxPA, Carcinoma ex pleomorphic adenoma; ACCa, adenoid cystic carcinoma; EMC, epithelial-myoepithelial carcinoma; SDC, salivary duct carcinoma; ANOS, adenocarcinoma not otherwise specified, poorly differentiated; MEC, mucoepidermoid carcinoma; LEC, lymphoepithelial carcinoma; SCCa, small cell carcinoma. ADC histogram values are expressed as mean ± s.d. ADC values (mean, median, and pADC threshold values) are expressed in ×10^-3^mm^2^/s.

¶, DOC, died of cancer: ACCa, lung metastasis (n = 1); SDC, lung/bone/liver metastasis (n = 1), neck recurrence (n = 1); ANOS, lung metastasis (n = 1); MEC, lung metastasis/neck recurrence (n = 1); SCCa, liver metastasis/abdominal lymph node metastasis/neck recurrence (n = 1).

The primary sites of the salivary gland cancers included parotid gland (n = 5), palatine (4), submandibular gland (3), buccal mucosa (2), sublingual gland (2), tongue base (1), parapharyngeal space (1), retromolar pad (1), and maxillary sinus (1). Histological subtypes of the 20 patients were summarized in **Table 1**. TNM classification was performed according to the UICC classification system [14]. The study protocol was approved by the Nagasaki University Ethics Committee (13040159), and the requirement to obtain informed consent for the review of images and records was waived for the retrospective nature of the study.

Status of surgical margins at the initial surgery of primary tumors, additional treatments received after the initial surgery, and prognoses of the 20 patients are summarized in **Table 2**.

**Table 2:** Status of surgical margins, additional treatment after the initial surgery, and prognosis of 20 patients with salivary gland carcinoma.

surgical margin¶	n	additional treatments after the initial surgery‡	n	prognosis	
				survived	DOC
negative	14	none	12§	9	3
		radiotherapy	1	1	0
		chemotherapy	1	0	1
positive	6	none	1†	0	1
		radiotherapy	1	1	0
		chemotherapy	2	2	0
		chemoradiotherapy	2	1	1
total	20		20	14	6

¶, histological evidence for the presence (positive) or absence (negative) of invasive tumor at the margin of resection.

‡, The treatments included chemo- and/or radiotherapy that were performed after the initial surgery.

§, These patients did not receive any additional treatment before recurrence occurred. Patients with recurrence had been treated by combinations of radiotherapy and chemotherapy.

†, This patient did not receive chemoradiotherapy due to severely impaired respiratory function.

DOC, died of cancer

### Conventional MR imaging

**Fig 1:**
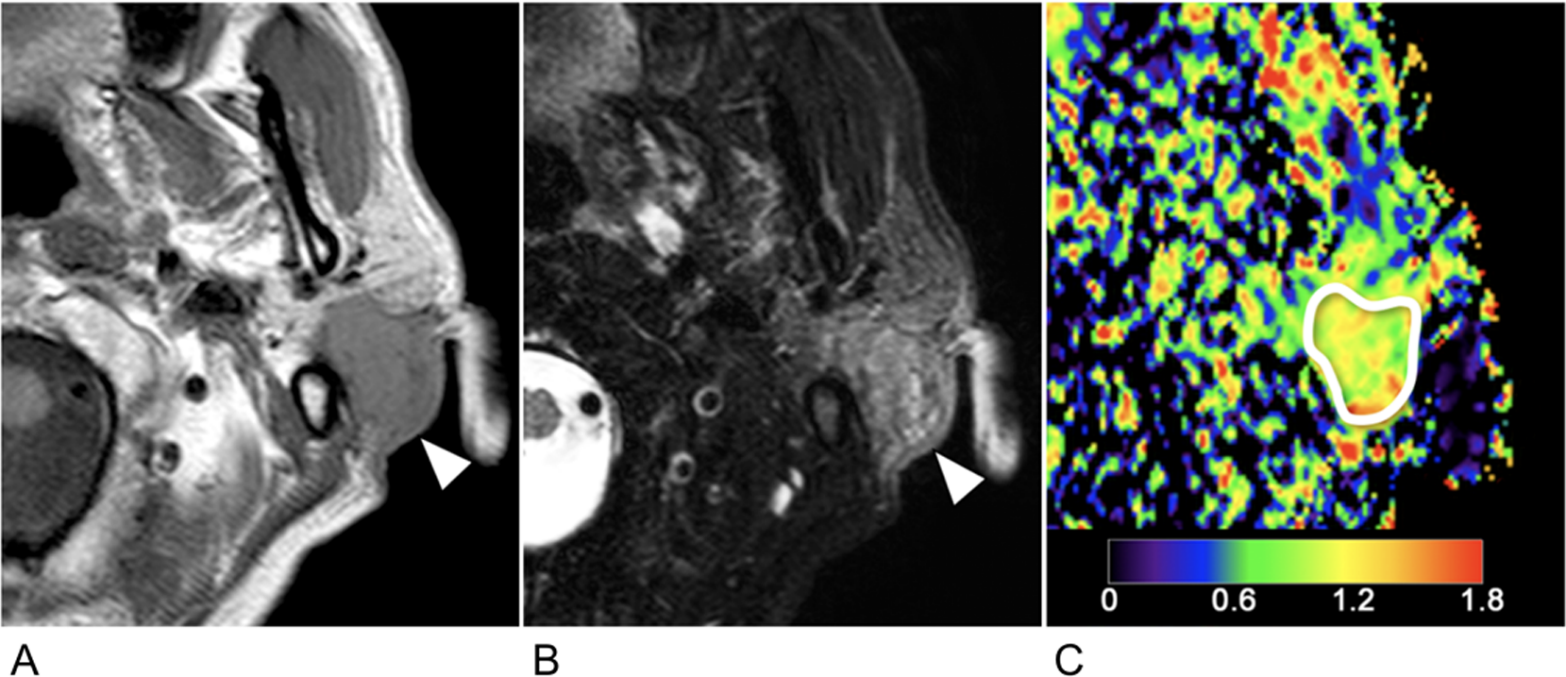
67-year-old man with mucoepidermoid carcinoma (T4N0M0). **A**, Axial T1-weighted MR image shows homogeneous and invasive cancer arising in left parotid gland (arrowhead). **B**, Axial fat-suppressed T2-weighted MR image shows heterogeneous parenchyma (arrowhead). **C**, Axial, color ADC map. White demarcation indicates an ROI manually placed within the tumor area for ADC measurement. Color scale bar indicates ADC levels (0–1.8 × 10^−3^ mm^2^/s). He had survived beyond 3 years (77 months) after surgical excision of primary cancer.

**Fig 2:**
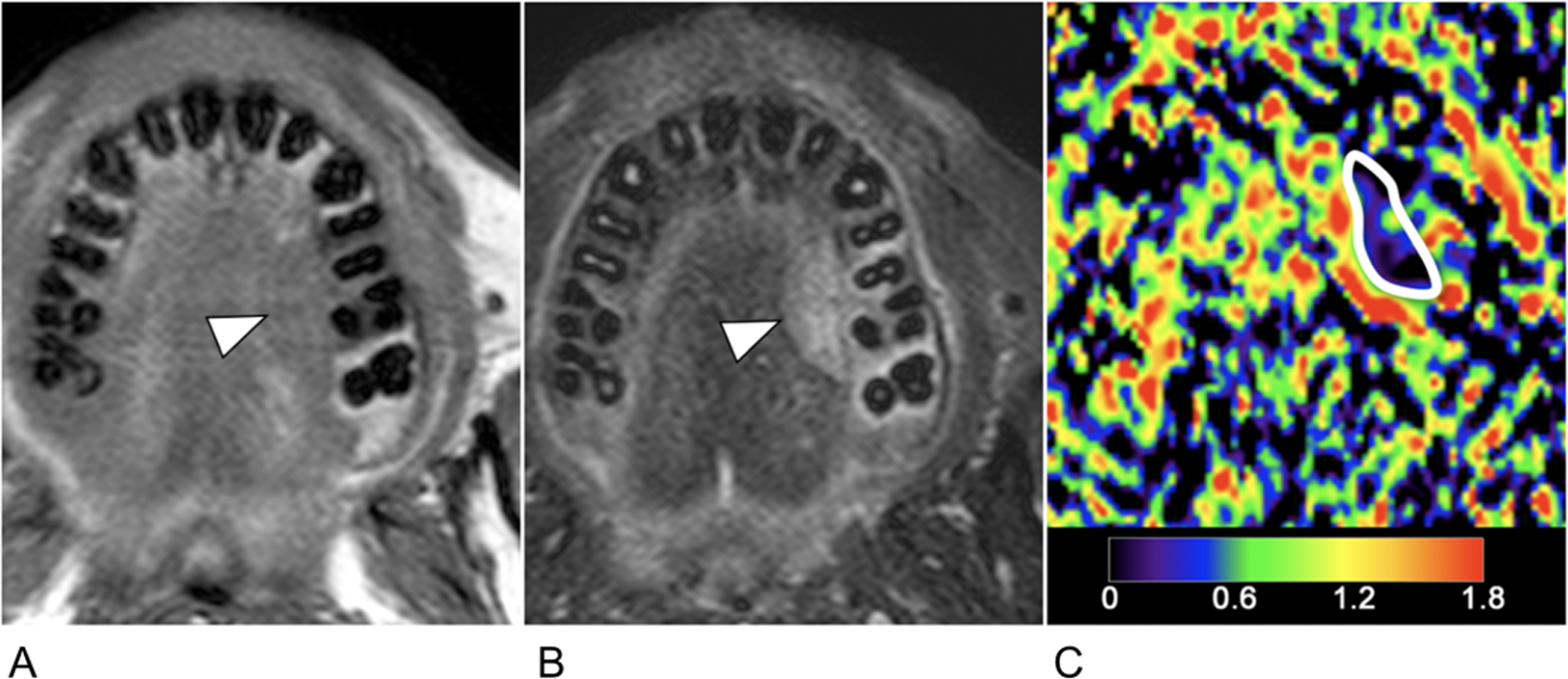
39-year-old man with small cell carcinoma (T3N1M0). **A**, Axial T1-weighted MR image shows ill-defined, homogeneous cancer arising in palatal gland (arrowhead). **B**, Axial fat-suppressed T2-weighted MR image shows heterogeneous parenchyma (arrowhead). **C**, Axial, color ADC map. White demarcation indicates an ROI manually placed within the tumor area for ADC measurement. Color scale bar indicates ADC levels (0–1.8 × 10^−3^ mm^2^/s). He died of cancer within the first 3 years (14 months) after surgical excision of primary cancer.

MR imaging was performed using a 1.5-T MR imager (Gyroscan Intera 1.5T Master, Philips Healthcare, Best, the Netherlands) with a 17 × 14-cm (Synergy-Flex M), 20 cm (Synergy-Flex L) surface coil, or a head and neck coil (Synergy Head Neck; Philips Healthcare). T1- and fat-suppressed (spectral attenuated with inversion recovery, SPAIR) T2-weighted MR images (TR/TE/number of signal acquisition = 500 ms/15 ms/2, and 6385 ms/80 ms/2, respectively) were obtained by using a turbo spin-echo (TSE) sequence (TSE factor = 3 and 15, respectively) (**Figs 1A, 1B**, **2A** and **2B**). We used a 200-mm field-of-view (FOV), 256 × 204 acquisition and 512 × 512 reconstruction matrix sizes, a 4-mm slice thickness and a 0.4-mm slice gap.

### Diffusion-weighted MR imaging

Axial diffusion-weighted images (TR/TE/numbers of signal acquisition = 4283 ms/87 ms/4) were obtained using single shot, spin-echo (SE) echo planar imaging (EPI), using b-values of 0, 500, and 1000 s/mm^2^. Isotropic diffusion images were obtained by applying the two higher b-values along the three orthogonal directions using a 200 × 200 mm^2^ FOV, 4-mm slice thickness, 0.4-mm slice gap and 112 × 90 matrix size. Phase-encoding was applied along the antero-posterior direction of the patients. The parallel imaging (sensitivity encoding, SENSE; SENSE factor = 2) technique was used for rapid image acquisition and for reduction of susceptibility artifact. Imaging time was 2 min and 8 s for the acquisition of 25 slices.

### ADC histogram analysis

ADC maps were obtained using b-values of 500 and 1000 s/mm^2^. Sequential gray-scale ADC map images that spanned the whole tumor lesion, except for the uppermost and lowermost slices, were saved in the digital imaging communication in medicine (DICOM) format; 1–9 slices were analyzed depending on the tumor sizes. A region of interest (ROI) along the inner margin of the tumor area was manually placed on the ADC map using the corresponding T1-weighted (with or without contrast-enhancement) and fat-suppressed T2-weighted MR images as references for placing the ROI (**Figs 1** and **2**). All the ADC maps from each cancer were analyzed by characterizing the ADC histogram profiles using (a) mean, (b) median, (c) percentage tumor area with specific ADC levels (pADC), (d) skewness, and (e) kurtosis. A radiologist with 21 years of experience in head and neck radiology read and analyzed all the MR images.

For assessing the pADC, tumor areas were classified into 4 categories on a pixel-by-pixel basis: extremely low (<0.6 × 10^−3^ mm^2^/s), low (0.6 × 10^−3^ mm^2^/s ≤ ADC <1.2 × 10^−3^ mm^2^/s), intermediate (1.2 × 10^−3^ mm^2^/s ≤ ADC <1.8 × 10^−3^ mm^2^/s), and high (≥1.8 × 10^−3^ mm^2^/s). The tumor areas with either one of these 4 categories were expressed as percentages of the total tumor area.

The ADC histograms were also analyzed by calculating histogram skewness and kurtosis; skewness and kurtosis were defined as *E*(*x* - *μ*)^3^/*σ*^3^ and *E*(*x* - *μ*)^4^/*σ*4–3, respectively, where E is the expected value, *μ* is the mean of *x*, and and *σ* is the standard deviation of *x*. A histogram with a normal distribution has a skewness of 0 and a kurtosis of 0. The skewness becomes more positive when a whole histogram shifts to the left as the frequency of low ADC values increased, the histogram peak shifted to the left, and the left tail of the histogram shortened. A higher kurtosis indicates that the histogram has a more acute peak, while a histogram with a lower kurtosis has a more flattened peak.

### Cross-validation

The diagnostic accuracy of the pADC was assessed by using leave-one-out cross-validation, where all the data except for a single case were used for training in each of 20-fold cross-validations.

### Statistical analysis

Univariate Cox proportional hazards regression models were used to assess the significance of various ADC histogram, TNM classification, age parameters, status of surgical margins, and additional treatments including chemotherapy and radiotherapy after the initial surgery in explaining patient survival during the entire follow-up period. Unadjusted hazard ratios (HRs) were calculated for each of the ADC parameters. Multivariable Cox-proportional hazard regression model was not available due to the small (n = 20) study population.

Univariate logistic regression analysis combined with receiver operating characteristic (ROC) curve analysis were performed to determine the relationship between the ADC parameters and the cancer deaths during the first 3 years after primary surgery. Variables with p values of <0.05 at the univariate regression analysis were subsequently assessed using an ROC analysis and the areas under the curves (AUCs) were compared among the variables.

Cohen’s d for the ADC histogram parameters between the DOC and survived patients was calculated according to the following formula:

$$d=(M_{DOC}-M_{survive})/{SD}_{pooled}$$ (1)

$${SD}_{pooled}=\sqrt{{[SD}_{DOC}^{2}(N_{DOC}-1)+{SD}_{survive}^{2}(N_{survive}-1)]/{(N}_{DOC}+N_{survive}-2)}$$ (2)

where SD_pooled_ is a pooled standard deviation, M_DOC_ and M_survive_ are respectively means for DOC and survived patients, SD_DOC_ and SD_survive_ are respectively standard deviations for DOC and survived patients, and N_DOC_ and N_survive_ are respectively numbers of DOC and survived patients.

The Cox proportional hazard regression, logistic regression, and ROC analyses were performed using JMP Pro (SAS, version 13) software. The cross-validation was performed using MATLAB software (MathWorks, version 2017a).

## Results

### ADC parameters relative to histological subtypes

**Fig 3:**
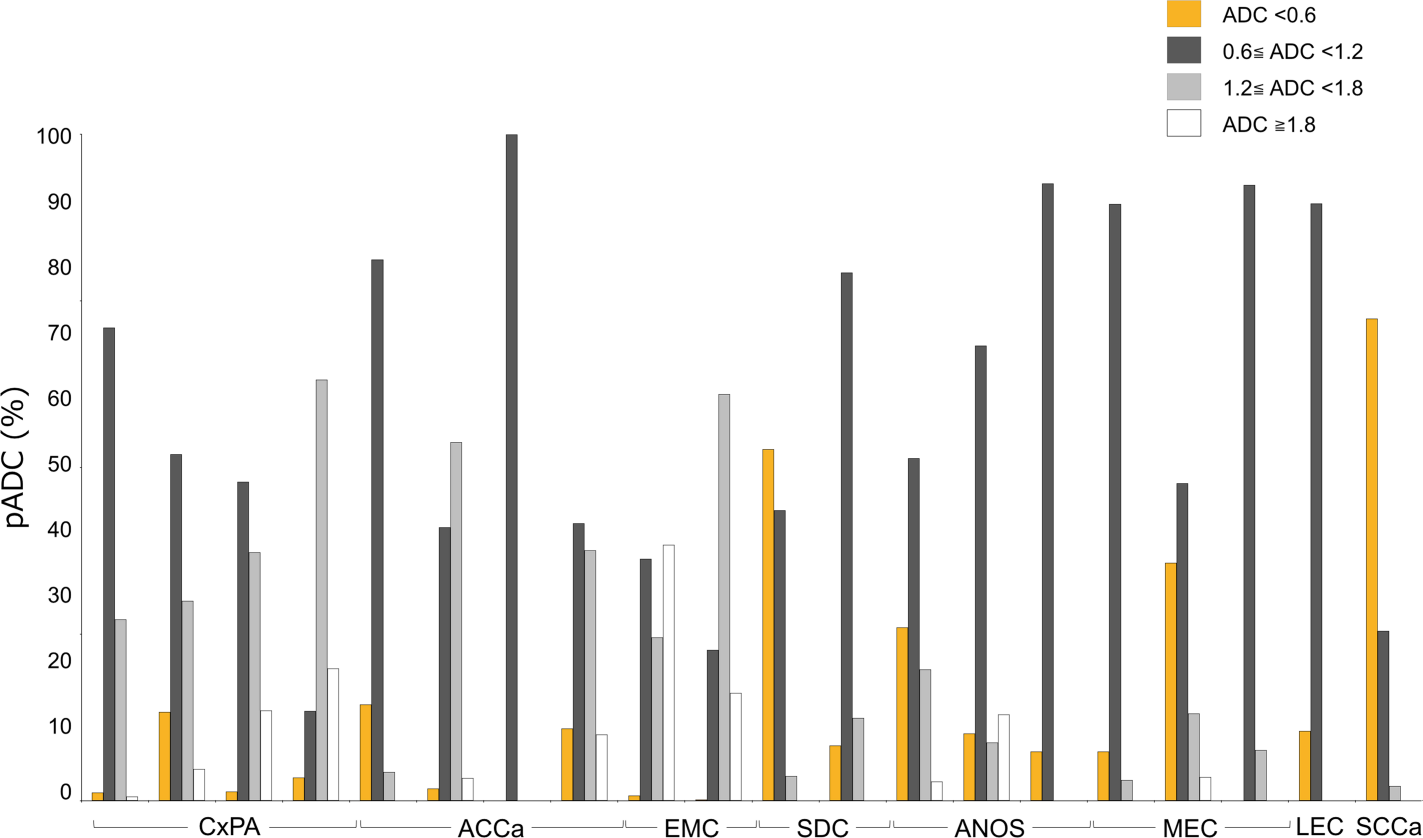
Distributions of percentage tumor areas with distinctive ADC values (pADC) of different types of salivary gland carcinomas. Bar graph shows pADCs of the 20 patients with salivary gland carcinoma. A whole tumor area was categorized into either of pADC with extremely low (<0.6 × 10^−3^ mm^2^/s), low (0.6 × 10^−3^ mm^2^/s ≤ ADC <1.2 × 10^−3^ mm^2^/s), intermediate (1.2 × 10^−3^ mm^2^/s ≤ ADC <1.8 × 10^−3^ mm^2^/s), or high (≥1.8 × 10^−3^ mm^2^/s) ADCs, and expressed as percentage tumor areas. CxPA, carcinoma ex pleomorphic adenoma; ACCa, adenoid cystic carcinoma; EMC, epithelial-myoepithelial carcinoma; SDC, salivary duct carcinoma; ANOS, adenocarcinoma not otherwise specified; MEC, mucoepidemoid carcinoma; LEC, lymphoepithelial carcinoma; SCCa, small cell carcinoma.

As expected, ADC histogram profiles differed greatly among cancers with different histological subtypes and also among cancers with the same histological subtypes (**Table 1**, **Fig 3**). Cancer death within the first 3 years after the primary surgery occurred in patients with salivary duct carcinoma (n = 2), adenoid cystic carcinoma (n = 1), adenocarcinoma not otherwise specified (n = 1), mucoepidermoid carcinoma (n = 1), or small cell carcinoma (n = 1). We found significant differences in ADC means (p = 0.049) and pADC <0.6 ×10^−3^ mm^2^/s (p = 0.034) between the histograms of patients who died of cancer (DOC) within the first 3 years after the primary surgery (n = 6) and those who had survived beyond the 3 years (n = 14) (**Table 3**).

**Table 3:** Comparison of ADC parameters between patients who died of cancer and those who were alive at the end of the follow-up periods.

	DOC¶	survived	p value§
ADC parameters	(mean ± s.d.)	(mean ± s.d.)	
mean (×10^−3^ mm^2^/s)	0.81 ± 0.26	1.10 ± 0.28	**0.049**
median (×10^−3^ mm^2^/s)	0.78 ± 0.28	1.05 ± 0.25	0.080
percentage tumor area with‡			
<0.6 ADC	34.3 ± 24.8	5.1 ± 5.2	**0.034**
0.6≤ADC<1.2	48.2 ± 17.7	64.1 ± 28.5	0.148
1.2≤ADC<1.8	14.8 ± 12.9	22.9 ± 23.3	0.335
≥1.8 ADC	2.7 ± 3.9	7.8 ± 11.3	0.152
skewness	0.58 ± 0.29	0.25 ± 0.58	0.112
kurtosis	1.14 ± 0.95	0.20 ± 0.78	0.066
UICC TNM classification	no. of patients	no. of patients	
T classificaton			0.430
Tis	0	2	
T2	0	4	
T3	2	3	
T4	4	5	
N classification			**0.016**
N0	0	12	
N1	1	0	
N2b	1	2	
N3b	3	1	
M classification			0.521
M0	5	13	
M1	1	1	
age	(mean ± s.d.)	(mean ± s.d.)	
	62 ± 16	60 ± 12	0.773
status of surgical margins	no. of patients	no. of patients	1.000
negative	4	10	
positive	2	4	
additional treatments	no. of patients	no. of patients	1.000
none	4	9	
radio- and/or chemotherapy	2	5	

¶, DOC, patients who died of cancer (DOC) within the first 3 years after the primary surgery; survived, patients who survived beyond 3 years after the primary surgery.

§, Welch’s test for ADC histogram parameters and ages; or Fisher’s exact test for UICC TNM classification grades, status of surgical margins, and additional treatments.

‡, ADC thresholds are expressed in ×10^−3^ mm^2^/s.

Age variables did not meet the proportional hazard assumption and thus were excluded from the analysis. Bold p values indicate statistically significant differences.

### TNM classification

TNM classification profiles of the patients are also summarized in **Table 3**. N category distributions were significantly different between the 2 patient groups with different outcomes (DOC during the first 3 years from the primary surgery vs. survivors beyond 3 years; p = 0.016), but not for the T- and M-category distributions.

### Status of surgical margins and additional treatments after the initial surgery

Distributions of the status of surgical margins at the initial surgery of primary tumors and the presence or absence of additional treatments after the initial surgery were not significantly different between the survived and DOC groups (**Table 3**).

### Correlation between the ADC parameters and patient survivals

Given the implications of some ADC histogram parameters and N-categories for the patient outcomes, we asked whether these variables could explain the outcomes of patients with salivary gland carcinoma throughout the follow-up periods (5–136 months). Univariate Cox-proportional hazards regression models indicated that mean ADC (unadjusted hazard ratio = 0.71, p = 0.034), median ADC (0.73, p = 0.048), pADC <0.6 ×10^−3^ mm^2^/s (1.05, p = 0.007), ADC kurtosis (2.88, p = 0.023), and N classification (2.24, p = 0.012) were significantly important in predicting patient outcomes (**Table 4**).

**Table 4:** Univariate Cox-proportional hazard regression models for the correlations between ADC parameters and the survival of patients with salivary gland carcinoma.

	unadjusted hazard ratio			p value
		per	95% CI	
ADC parameters				
mean	0.71	1 × 10^−4^ mm^2^/s	0.494–0.977	**0.034**
median	0.73	1 × 10^−4^ mm^2^/s	0.511–0.997	**0.048**
percentage tumor area with‡				
<0.6 ADC	1.05	1%	1.013–1.082	**0.007**
0.6≤ADC<1.2	0.98	1%	0.949–1.013	0.262
1.2≤ADC<1.8	0.98	1%	0.930–1.022	0.400
≥1.8 ADC	0.93	1%	0.749–1.032	0.216
skewness	2.40	1	0.572–8.869	0.221
kurtosis	2.88	1	1.166–7.420	**0.023**
UICC TNM classification				
T classification†	2.79	1 grade	0.998–15.28	0.051
N classification¶	2.24	1 grade	1.196–4.836	**0.012**
M classification§	2.19	1 grade	0.114–13.822	0.513
age	1.29	≥60 vs <60 years	0.239–6.998	0.754
status of surgical margins	1.38	positive vs negative	0.192–7.107	0.713
additional treatments	1.00	none vs performed	0.195–7.204	0.999

Hazard ratios are expressed per indicated units. 95% CI, 95% confidence interval.

‡, ADC thresholds are expressed in × 10^−3^ mm^2^/s.

†, n = 2, 4, 5, 9 for T1s, T2, T3, and T4

patients, respectively.

¶, n = 12, 1, 3, and 4 for N0, N1, N2b, and N3b patients, respectively.

§, n = 18 and 2 for M0 and M1 patients, respectively.

Age variables did not meet the proportional hazard assumption and thus were excluded from the analysis. Bold p values indicate statistically significant differences (p <0.05).

As expected, there were no significant relationships between the status of surgical margins or additional treatments after the initial surgery and the patient survivals (**Table 4**).

### Correlations between the ADC parameters and cancer death

Next, we assessed the correlations of the ADC parameters, TNM classification profiles, age distributions, status of surgical margins, and additional treatments after the initial surgery with the cancer death of the 6 patients that occurred within the first 3 years after the primary surgery. Univariate logistic regression analysis indicated that mean ADC (unadjusted odds ratio = 0.62, p = 0.026), median ADC (0.64, p = 0.038), pADC <0.6 ×10^−3^ mm^2^/s (1.28, p<0.001), ADC kurtosis (4.73, p = 0.023), and N category (2.79, p = 0.014) were significantly correlated with cancer death of the 6 patients (**Table 5**).

**Table 5:** Univariate logistic regression and ROC analyses for the correlations between ADC parameters and cancer death in 20 patients with salivary gland carcinoma.

	univariate logistic regression analysis				ROC analysis	
	unadjusted odds ratio§		95% CI	p value	cutoff threshold†	AUC
		per				
ADC parameters						
mean	0.62	1 × 10^−4^ mm^2^/s	0.357–1.058	**0.026**	≤9.44 × 10^−4^ mm^2^/s	0.762
median	0.64	1 × 10^−4^ mm^2^/s	0.382–1.066	**0.038**	≤7.56 × 10^−4^ mm^2^/s	0.738
percentage tumor area with‡						
<0.6 ADC	1.28	1%	0.964–1.686	**<0.001**	≥8.3%	0.929
0.6≤ADC<1.2	0.97	1%	0.935–1.016	0.195		
1.2≤ADC<1.8	0.98	1%	0.926–1.033	0.395		
≥1.8 ADC	0.92	1%	0.771–1.087	0.214		
skewness	3.81	1	0.453–31.996	0.186		
kurtosis	4.73	1	0.846–26.394	**0.023**	≥0.61	0.798
UICC TNM classification						
T classification*	3.25	1 grade	0.795–13.913	0.054		
N classification**	2.79	1 grade	1.124–6.942	**0.014**	≥N1	0.821
M classification***	2.60	1 grade	0.135–50.049	0.531		
age	1.33	≥60 vs <60 years	0.196–9.083	0.769		
status of surgical margins	1.25	positive vs negative	0.160–9.765	0.832		
additional treatments	1.11	none vs performed	0.148–8.367	0.919		

§, Odds ratios are expressed as those per indicated units. 95% CI, 95% confidence interval.

†, These cutoff thresholds yielded 83% sensitivity, 64% specificity, 70% accuracy for ADC mean; 50% sensitivity, 93% specificity, and 80% accuracy for ADC median; 100% sensitivity, 71% specificity, and 80% accuracy for pADC <0.6 ×10^−3^ mm^2^/s; and 83% sensitivity, 71% specificity, and 75% accuracy for ADC kurtosis; 83% sensitivity, 79% specificity, 80% accuracy for N classification. AUC, area under curve

‡, ADC thresholds are expressed in ×10^−3^ mm^2^/s.

*, n = 2, 4, 5, 9 for T1s, T2, T3, and T4 patients, respectively.

**, n = 12, 1, 3, and 4 for N0, N1, N2b, and N3b patients, respectively.

***, n = 18 and 2 for M0 and M1patients, respectively. Age variables did not meet the proportional hazard assumption and thus were excluded from the analysis. Bold p values indicate statistically significant differences (p <0.05).

The small study cohort of the present study did not allow for a multivariable analysis of the variables that were independently significant in predicting cancer death. Therefore, we performed a ROC analysis to evaluate the predictive ability of the ADC histogram and TNM classification variables that were significantly important in the univariate logistic regression models. The ROC analysis indicated that pADC <0.6 ×10^−3^ mm^2^/s (AUC = 0.929; cutoff threshold ≥8.3%) was the best tested parameters for predicting cancer death in patients with salivary gland cancer (**Table 5**). Leave-one-out cross-validation analysis (k = 20) indicated that the predictive accuracy using the threshold of pADC <0.6 ×10^−3^ mm^2^/s was 90%.

We further evaluated the significance of differences in the ADC histogram parameters between the DOC and survived patients by calculating Cohen’s d. The d values between the DOC and survived patients for the ADC mean, ADC median, pADC with extremely low ADC, and kurtosis were 1.06, 1.04, 2.12, and 1.13, respectively.

## Discussion

In the present study, we have shown that the ADC histogram analysis can effectively predict the outcomes of patients with salivary gland carcinoma. The mean ADC, median ADC, pADC with extremely low (<0.6 ×10^−3^ mm^2^/s) ADC levels, ADC kurtosis, and N classification grade were variables that significantly correlated with the patient outcomes in both the univariate Cox proportional hazard and univariate logistic regression models. However, the pADC with extremely low (<0.6 ×10^−3^ mm^2^/s) ADC levels was the best variable for predicting cancer death.

Salivary gland carcinomas have heterogeneous histological architectures, composed of cancer nests with different cell sizes and densities and of extracellular components with different tissue types and viscosities [15]. Therefore, carcinomas of different histological subtypes and even of the same histological subtypes can display divergent ADC histogram profiles depending on the varying areas of histological components. For example, cancer nests composed of large epidermoid or polygonal clear cancer cells may have intermediate ADC vales, while those composed of densely packed, poorly differentiated cancer cells may have low ADC values [1, 15]. By contrast, large areas of necrotic tissues and cystic/sinusoid cavity in salivary gland carcinomas may be associated with high ADC values. Accordingly, an overall ADC measurement of the whole tumor area would mask relatively small cancer areas that might have ADC values characteristic of the aggressiveness and sensitivity to treatment of the cancer [16–18]. In this regard, assessing the pADC, which indicates percentage tumor areas with distinctive tissue-specific ADC levels [15], would be a better strategy for characterizing salivary gland carcinomas. In support of this notion, previous studies showed that the differential profiling of pADC effectively discriminated salivary gland cancers from benign salivary gland tumors [8, 19]. This technique was also effective for differentiating between some histological types of benign salivary gland tumors (i.e., pleomorphic adenomas vs, Warthin’s tumors), but not for the differentiation between histological subtypes of salivary gland carcinomas.

The general consensus is that some histological subtypes of salivary gland carcinoma are aggressive with a poor prognosis, while others are less aggressive with an excellent prognosis [6]. However, different carcinomas with the same histological subtype may have distinct prognoses associated with a variable growth potential, degree of cancer extension beyond the capsule, and patterns of specific protein expression, such as Ki-67, epidermoid growth factor receptor (EGFR), HER2, c-kit, and vascular endothelial growth factor (VEGF) [1, 6]. Therefore, we should predict the prognosis of patients with salivary gland carcinoma using different prognostic markers, irrespective of the histological subtypes. To this end, a proper imaging technique would be beneficial to an effective pre-treatment prediction of patient outcomes. The present study has shown that the pADC could be used for this end. Currently, we have no definite idea of how the increased values of pADC with extremely low ADC fraction contributed to the patient prognosis. A possible explanation may be that cancer nests with extremely low ADC values have the potential for rapid and aggressive cancer growth. Consistent with this idea, a close relationship was found between low ADC values and expression levels of Ki-67 protein, which is considered a molecular marker of accelerated cell proliferation [20, 21]. Furthermore, a high level of Ki-67 protein expression was reportedly associated with high mortality of patients with various subtypes of salivary gland carcinomas [16, 22].

Previous studies showed that the ADC histogram analysis using skewness and kurtosis was highly predictive of histological grades and treatment efficacy of head and neck squamous cell carcinomas [11, 12]. However, in the present study, we found that these ADC histogram parameters were less effective than the pADC in predicting cancer death of patients with salivary gland carcinoma. Roughly consistent with the present results, Dinh et al. showed that the lower 10th percentile of the ADC was a potent indicator of aggressive prostate cancers [13]. Histological and biological interpretation for the magnitudes of ADC skewness and kurtosis may be puzzling. Considering that the ADC skewness and kurtosis was not significantly correlated with the pADC [<0.6 ×10^−3^ mm^2^/s] parameter (*r* = 0.35 for skewness and *r* = 0.38 for kurtosis, Pearson’s correlation coefficients), the ADC skewness and kurtosis may not be directly correlated with the presence or absence of densely packed small cancer cells within the tumor. Furthermore, the analysis of ADC percentile may be greatly affected by the mean of the ADC histogram. In this regard, the pADC criterion could be used as a separate indicator for predicting the outcomes of patients with salivary gland carcinoma.

A previous study showed that an advanced N classification was the only significant predictor of the survival of patients with salivary gland carcinoma [23]. In the present study, we also found that N category (≥N1) was a risk of cancer death of the patients. However, this parameter was much less predictive of cancer death when compared to that of the pADC [<0.6 ×10^−3^ mm^2^/s] variable. Six patients in this study cohort died of cancer after distant metastasis and/or neck recurrence. Therefore, the growth potential of metastatic foci in the regional nodes and/or in the primary lesions may more strongly influence the patient outcomes rather than nodal metastasis per se. Related to this notion, lymph node size is considered critical for predicting distant metastasis [24]. Furthermore, the presence of extranodal spread (ENS) increases the chance of local failure and distant metastasis [25–27]. Elevated expressions of hypoxia-inducible factor α (HIF-α) in cancer foci with low ADC values might explain the molecular basis for aggressiveness of the cancer in the primary and metastatic lesions of salivary gland carcinomas [21].

The status of surgical margins has been considered important for the prognosis of patients with head and neck cancers including salivary gland carcinomas, and histological confirmation of a positive margin would require adjuvant therapy [28, 29]. In the present study cohort, all the patients with positive margins had received adjuvant chemo- and/or radiotherapy, except for one who was associated with severely impaired respiratory function at the initial surgery, and there was no significant relationship between the status of surgical margins and the prognosis of patients. Although there is no prospective, controlled evidence to support the use of adjuvant treatments for positive surgical margins in patients with head and neck cancers [28], the presence of positive surgical margins may not greatly affect the prognosis of patients with salivary gland carcinoma if complete eradication of the residual tumor or appropriate adjuvant therapy is conducted.

A major limitation of this study is obviously the small study cohort. This shortcoming did not allow us to determine the factors that significantly and independently influenced the outcomes of the patients with salivary gland carcinoma. In addition, the spectrum of histological subtype was narrow and the results may be biased accordingly. Most of the variables tested in the present study were not closely related to the others. Therefore, multivariable Cox proportional hazard models and multivariable logistic regression analysis could define the important and independent ADC histogram parameters for predicting the patient outcome in a large cohort of patients with a wide histological spectrum. Salivary gland carcinomas are very rare. In addition, ADC calculation is very sensitive to the MR sequences used; therefore, diffusion-weighted imaging using the same imaging parameters is required for reliable comparison of ADC values between different patients. Therefore, a prospective, multicentric study using the same type of MR machine should be conducted for obtaining the conclusive results regarding the importance of the ADC histogram analysis for predicting patient outcomes. This could be a very tough project. In this regard, high Cohen’s d values (>1.0) of the ADC histogram parameters (ADC mean, ADC median, pADC with extremely low ADC, and kurtosis) imply the predictive potential of the parameters in a large patient cohort. Another limit may be that the present study was conducted within a very narrow disease entity of head and neck cancer, thus whether the pADC variables are also useful for predicting cancer death/outcome in other types of head and neck cancer is unknown. Positive results from a recent study on the predictive ability of ADC percentile variables for treatment outcomes in patients with head and neck squamous cell carcinomas are very encouraging [30].

In conclusion, ADC histogram analysis may be helpful for the prediction of cancer death risk in patients with salivary gland carcinoma. Specifically, the pADC [<0.6 ×10^−3^ mm^2^/s] variable is more predictive of cancer death compared with ADC mean, median, skewness, or kurtosis.
